# Ultra-Small Silver Nanoparticles: A Sustainable Green Synthesis Approach for Antibacterial Activity

**DOI:** 10.3390/antibiotics12030574

**Published:** 2023-03-14

**Authors:** Javier Emanuel Castañeda-Aude, José Rubén Morones-Ramírez, David Alejandro De Haro-Del Río, Angel León-Buitimea, Enrique Díaz Barriga-Castro, Carlos Enrique Escárcega-González

**Affiliations:** 1 Universidad Autónoma de Nuevo León, Facultad de Ciencias Químicas, Pedro de alba, s/n, San Nicolás de los Garza 66455, Nuevo León, Mexico; 2 Universidad Autónoma de Nuevo León, Facultad de Ciencias Químicas, Centro de Investigación en Biotecnología y Nanotecnología, Parque de Investigación e Innovación Tecnológica, Apodaca 66629, Nuevo León, Mexico; 3 Centro de Investigación de Química Aplicada, Enrique Reyna H. No. 140, San José de los Cerritos, Saltillo 25294, Coahuila, Mexico

**Keywords:** ultra-small nanoparticles, clinical multidrug-resistant bacteria, silver nanoparticles, green synthesis, organic waste

## Abstract

The present study centers on the synthesis of ultra-small silver nanoparticles (AgNPs) with antibacterial properties using citrus peel residues (orange, lemon, and grapefruit) as reducing and stabilizing agents, and on assessing their antibacterial activity against multidrug-resistant clinical *Staphylococcus aureus*. The synthesized AgNPs were analyzed by various techniques, including UV-Vis spectroscopy, SAED, TEM, XRD, FTIR, and Raman. The results demonstrate the formation of ultra-small, monodisperse, quasi-spherical AgNPs with an average particle size of 2.42 nm for AgNPs produced with mixed extracts. XRD analysis indicated that the AgNPs have a crystal size of 9.71 to 16.23 nm. The AgNPs exhibited potent inhibitory activity against resistant *S. aureus*, with a minimum inhibitory concentration (MIC) of 15.625 to 62.50 ppm. The findings suggest that the ultra-small nanometer size of the AgNPs could be attributed to the synthesis method that employs ambient conditions and the presence of polyphenolic compounds from citrus peel. Consequently, AgNPs obtained through sustainable green synthesis hold significant potential in combating clinical multi-resistant bacterial strains that are challenging to treat and eradicate. This approach also contributes to the revaluation of citrus residues in the region, which is an ongoing environmental issue today.

## 1. Introduction

The use of nanomaterials in biomedical applications is becoming increasingly popular, with metallic nanoparticles being derived from metals such as gold, silver, zinc, and iron due to their advantageous chemical and biological properties. To ensure the sustainability of this process, novel ecological and environmentally friendly approaches to synthesize these nanoparticles must be explored [1,2,3].

The inappropriate use of antibiotics has become a major global health concern as it has led to the emergence and spread of antibiotic-resistant bacteria. Due to inadequate access to antibiotics without a prescription, inadequate diagnosis of the underlying condition, and widespread and indiscriminate use of antibiotics, the threat of antibiotic resistance is increasing rapidly [4]. Examples of the above include methicillin-resistant *Staphylococcus aureus* [5], ampicillin- and kanamycin-resistant *S. aureus* [6], or multidrug-resistant *S. aureus* [7]. Therefore, there has been a growing interest in studies aimed at developing new antibacterial therapies, such as noble metal nanoparticles [8].

These nanoparticles can have a range of sizes from 1 to 100 nm, with a variety of morphologies such as spherical [9], triangular [10], and core-shell shapes [11]. Many of them have been applied in different areas taking advantage of their properties that thesematerial can have due to their nanometric size. In the fact, recently, nanomaterials have been used in biomedical applications against different species of bacteria such as *Escherichia coli* [12], *Staphylococcus aureus* [13], *Pseudomona aeruginosa* [14], and *Aspergillus flavus* [15], among others.

Lately, many sustainable AgNP synthesis routes have been reported using fruit and vegetable extracts such as *Acacia rigidula* [16], *Eriobotrya japonica* leaf [17], *Carica papaya* peel [18], cauliflower waste [19], *Rosa Chinensis* L. [20], *Morinda citrifolia* L. [21], banana peel [22], and walnut shell [23].

According to the Food and Agriculture Organization of the United Nations (FAO), in 2020, the total global production of citrus was 158 million tons. Processing one ton of oranges results in up to 0.6 tons of citrus solid waste, 0–9% of a ton of seeds, 60–75% of a ton of peels, and 23–33% of a ton of membrane waste [24].

To reduce the environmental problems of the final disposal of citrus waste such as lemon, orange, and grapefruit, extracts have been used for the synthesis of silver nanoparticles [25], as well as those derived from other metals like copper [26], zinc [27], platinum [28], and palladium [29]. This type of nanoparticles which can be ultra-small in size, have been used as antimicrobial agents.

In relation to this, different investigations of ultra-small metallic nanoparticles have found that these nanomaterials can easily pass through the pores of the cell wall of bacteria and can be internalized within them, disrupting their function and promoting the generation of reactive oxygen species (ROS) that cause the oxidation of the bacterial membrane, altering bacterial metabolism [30]. Furthermore, ultra-small nanoparticles of different metals such as gold [31], iron oxide [32], and copper [33] have recently been synthesized. Ultra-small AgNPs have been obtained by green synthesis using extracts of *Sida cordifolia* [34], Rosa ‘*Andeli*’ [35], and *Mentha aquatica* leaf [36]. Thus, although AgNPs have been synthesized with different organic material, the synthesis of ultra-small AgNPs in combination with different citrus fruits has yet to be reported.

The present study employed an extract of orange, lemon, grapefruit, and mixture (lemon, orange, grapefruit) peels to synthesize ultra-small AgNPs with potent antibacterial activity against a multi-drug resistant strain of *Staphylococcus aureus*. The extract was also found to possess antioxidant properties which enabled it to reduce silver metal ions. Thus, making use of extracts from orange, lemon, and grapefruit peels as reducing and stabilizing agents is an effective alternative for the fabrication of ultra-small antibacterial silver nanoparticles.

## 2. Materials and Methods

### 2.1. Sample Collection

*Citrus sinensis* (orange), *Citrus aurantifolia* (Mexican lemon), and *Citrus paradisi* (grapefruit) peels were collected from January to March in Nuevo León, Mexico. These peels were thoroughly washed with distilled water, cut to a size of 1 cm^2^. With respect to each of the three different peels, 20 g were weighed, and in the case of the mixture with three citrus peels, a total of 20 g was also obtained, consisting of 6.67 g of each of the peels. 

### 2.2. Preparation of Citric Peel Extract

Individual aqueous extracts were prepared for each citrus peel and the mixture. An amount of 20 g of the citrus peels, in combination, were added to 100 mL of distilled water in a beaker. The mixture was covered with aluminum and stored for 24 h at room temperature without shaking. Then, this mixture was filtered and stored at 4 °C for subsequent use.

### 2.3. Preparation of Silver Nanoparticles (AgNPs)

First, 20 mL of citrus peel extract was put into a 50 mL Corning tube. Then, 20 mL of an aqueous solution of 0.1 M silver nitrate was added to the tube. The tube was placed on a rack and left to react at room temperature for 24 h. During this time, the formation of AgNPs was observed by a color change in the mixture, which went from amber to brownish black. After 24 h, the particles were separated from the mixture through centrifugation using a Thermo Scientific Sorvail Legend XFR machine, which was set to 9000 rpm for 15 min. The separated particles were then filtered and the remaining residue was washed twice with 96% ethanol to get rid of any impurities and organic material. Finally, this process resulted in stable AgNPs.

### 2.4. Characterization of Nanoparticles

The AgNPs that were obtained underwent various types of analyses to determine their characteristics. UV-visible spectrophotometry was used to study them with a Thermo Scientific Multiskan GO machine. To assess the structural and morphological traits of the AgNPs, they were examined with transmission electron microscopy (TEM) and high-resolution TEM (HRTEM) utilizing the FEI-TITAN microscope that operates at an acceleration voltage of 300 kV. In addition, energy-dispersive spectrometry (EDS) was conducted to identify the elements present in the samples. The same TEM equipment was used to carry out selected area electron diffraction (SAED), while X-ray diffraction (XRD) analyses were performed on a PANalytical X’Pert Pro machine. A Fourier transform infrared (FT-IR) spectrophotometer (Spectrum One; Perkin Elmer, Waltham, MA, USA) was used to acquire IR-spectra. The Raman analysis was carried out in frequency range of 2000–200 cm^−1^ using a Micro-Raman Horiba XploRA instrument with a 532 nm laser.

### 2.5. Tested Bacteria

The gram-positive bacteria used in the antimicrobial trials were a multidrug-resistant clinical *Staphylococcus aureus* (a kanamycin-, chloramphenicol-, ciprofloxacin-, and ampicillin-resistant strain) isolated at the Hospital San Vicente de Monterrey, Nuevo León, Mexico.

### 2.6. Antimicrobial Activity of AgNPs

To culture the bacteria, the overnight method was used, which involved allowing the bacteria to grow for 17 h at 37 °C and 100 rpm using a Lab Companion IS-971.After the incubation period, the cultures were diluted by a factor of 1:100 in LB medium and then the inoculum (taken from the culture) was allowed to grow for 2–3 h. Subsequently, a sample of the bacterial culture was taken, adjusting with fresh medium until the optical density at 600 nm reached a reading of 0.2 ± 0.02 in a final volume of 1 mL, using OPTIZEN 2120UV measurement equipment. This resulting suspension was then diluted by a factor of 1:100 in LB medium to create the bacterial working solution.

MIC assays were conducted in 96-well plates. First, AgNPs stocks were prepared using LB medium as a vehicle, with a final concentration of 1 mg/mL. This stock of AgNPs was added to the 96-well plate until the concentration reached 250 μg/mL in a final volume of 200 μL. Serial dilutions of AgNPs were then performed by taking 100 μL of the previous solution to the next well, adding 100 μL of culture medium, and discarding the last 100 μL of the previous dilution. The concentrations of AgNPs tested ranged from 250–15.625 μg/mL. Next, 100 μL of the *S. aureus* solution was added to each well of the assay, and the 96-well plate was incubated at 37 °C. The optical density was measured every hour for a total of 23 h. The concentration at which bacterial growth was inhibited was defined as the MIC. All tests were performed in triplicate.

MIC tests for citrus peel extracts. A solution of 20 microliters of the extract (lemon, orange, grapefruit, or mixture) was prepared in 380 microliters of LB medium and the same procedure as for the MICs of AgNPs was followed.

## 3. Results

### 3.1. Characterization of AgNPs

**Figure 1:**
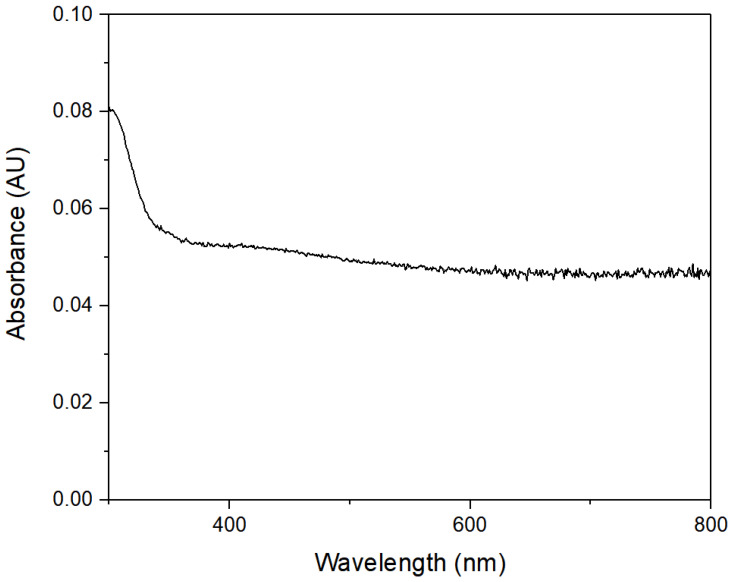
UV-vis absorbance spectrum of the control experiment: silver ions in deionised water.

**Figure 2:**
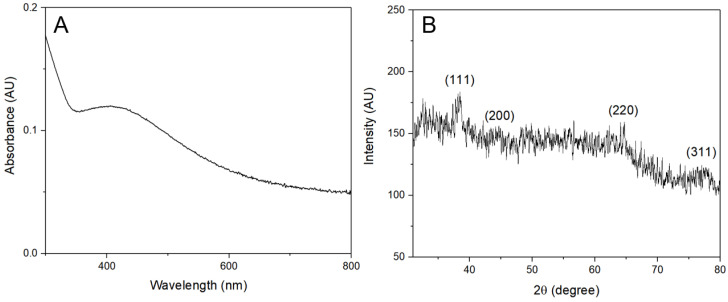
Characterization of the AgNPs synthesized with lemon peel extract. (**A**) UV-vis absorbance spectrum of the AgNPs. (**B**) XRD pattern of the AgNPs with labeled diffraction peaks.

**Figure 3:**
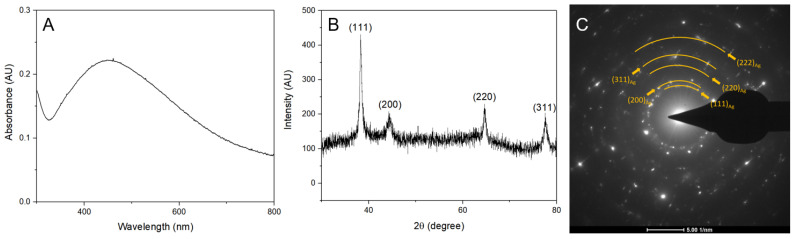
Characterization of the AgNPs synthesized with orange peel extract. (**A**) UV-vis absorbance spectrum of the AgNPs. (**B**) XRD pattern of the AgNPs with labeled diffraction peaks. (**C**) SAED pattern of the AgNPs with the rings labeled.

**Figure 4:**
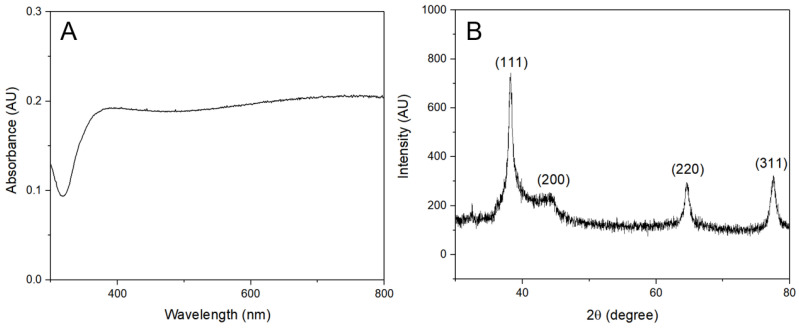
Characterization of the AgNPs synthesized with grapefruit peel extract. (**A**) UV-vis absorbance spectrum of the AgNPs. (**B**) XRD pattern of the AgNPs with labeled diffraction peaks.

**Figure 5:**
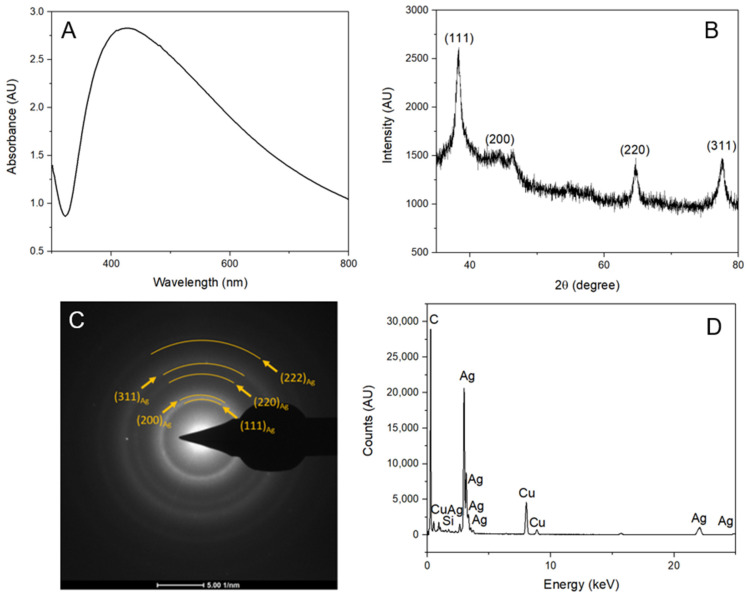
Characterization of the AgNPs synthesized with a mixture of lemon, orange, and grapefruit peels extract. (**A**) UV-vis absorbance spectrum of the AgNPs. (**B**) XRD pattern of the AgNPs with labeled diffraction peaks. (**C**) SAED pattern of the AgNPs with the rings labeled. (**D**). EDS spectrum of the AgNPs with the peaks labeled.

Figure 1 shows the UV-Vis spectrum of the control experiment where silver ions were kept in water, confirming that there was no reduction of the silver salt by itself or due. Figure 2A, Figure 3A, Figure 4A and Figure 5A confirm the presence of AgNPs produced using an aqueous solution containing extracts of orange, lemon, and grapefruit peel, and of their mixture. The AgNPs exhibit a plasmon resonance spectrum with an absorption band between 400 and 450 nm. The peak width of the AgNPs synthesized with grapefruit peel extract may be due to the variation in particle size. In summary, the UV-Vis spectrum provides quantitative data to confirm the presence of AgNPs in the samples.

Furthermore, the X-ray diffraction (XRD) pattern of the AgNPs synthesized with lemon peel extract is illustrated in Figure 2B and it displayed characteristic diffraction peaks at 38.44, 44.57, 64.68, and 70.02. Similarly, the AgNPs synthesized with orange peel (Figure 3B) and grapefruit peel (Figure 4B) extracts exhibited peaks at 38.24, 44.31, 64.63, and 77.53, and 38.22, 44.02, 64.57, and 77.55, respectively. The mixture of extracts (Figure 5B) showed peaks at 38.29, 44.62, 64.82, and 77.70. All these peaks corresponded to the (111), (200), (220), and (311) planes of face-centered cubic silver. It can be concluded that the AgNPs synthesized using the extracts of citrus peels possessed a crystalline structure similar to that of metallic silver, which further confirms the successful green synthesis of AgNPs. 

Figure 3C and Figure 5C depict the selected area electron diffraction (SAED) pattern of the AgNPs, which revealed well-defined rings that corresponded to the (111), (200), (220), (311), and (222) planes of face-centered cubic (fcc) metallic silver.

Figure 5D shows the characterization by EDS analysis that determines the presence of silver, confirming the presence of AgNPs. In this analysis, other elements such as Cu and C were detected, which correspond to the carbon lacey films on the copper TEM grid.

The Scherrer equation was used to calculate the crystal size of the synthesized AgNPs by analyzing the X-ray diffraction data. The equation considers the full width at half maximum (FWHM) of the peaks obtained from the XRD patterns, which corresponds to the size of the crystallites. The Bragg angle (θ) for the X-ray diffraction was 2θ = 38°, corresponding to the (111) plane of the face-centered cubic structure of silver.

(1) $$D\;=\frac{K\lambda }{\beta \cos\theta }$$

Scherrer’s constant, commonly taken as 0.9, and the wavelength of Cu K radiation (1.54 Å) were used in the Scherrer equation. The half maximum full width (β) was determined from the peak at 2θ = 38° for each sample. The average crystal size was obtained by calculating the value of D for each sample.

The crystal size of the silver nanoparticles synthesized with lemon, orange, grapefruit, and a mixture of these citrus peels extracts was determined using the Scherrer equation. The average crystal size of the AgNPs synthesized with lemon peel extract was found to be 9.71 nm. In the case of the AgNPs synthesized with orange peel extract, it was 16.23 nm. The crystal size for the extracts of grapefruit peel and for the combination was found to be 10.70 nm and 9.86 nm, respectively.

**Figure 6:**
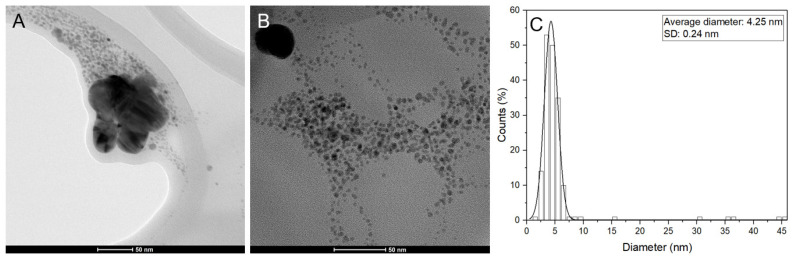
Characterization of the AgNPs synthesized with orange peel extract. (**A**) TEM micrograph of the AgNPs. (**B**) High-resolution TEM micrograph of the AgNPs. (**C**) Histogram showing the particle size distribution.

**Figure 7:**
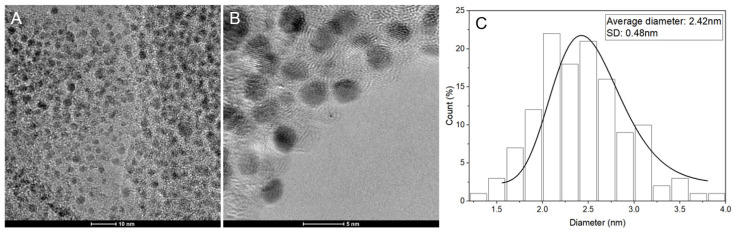
Characterization of the AgNPs synthesized with a mixture of lemon, orange, and grapefruit peels extract. (**A**) TEM micrograph of the AgNPs. (**B**) High-resolution TEM micrograph of the AgNPs. (**C**) Histogram showing the particle size distribution.

The morphology of the AgNPs was determined by TEM and HRTEM images (Figure 6 and Figure 7). These were obtained to analyze the size and shape of the particles, and histogram data were analyzed to determine the size distribution. The particles showed a spherical shape with a uniform distribution, and the average size of the particles ranged from 2.42 nm to 4.25 nm.

The TEM images for each extract showed a spherical shape with a narrow size distribution. The orange extract-synthesized nanoparticles (Figure 6) had an average size of 4.25 nm, deviation of 0.24 nm, and a positively skewed histogram indicating the presence of smaller nanoparticles. The mixed extract-synthesized nanoparticles (Figure 7) had an average size of 2.42 nm, deviation of 0.48 nm, and a normal distribution histogram indicating a relatively even distribution of sizes.

**Figure 8:**
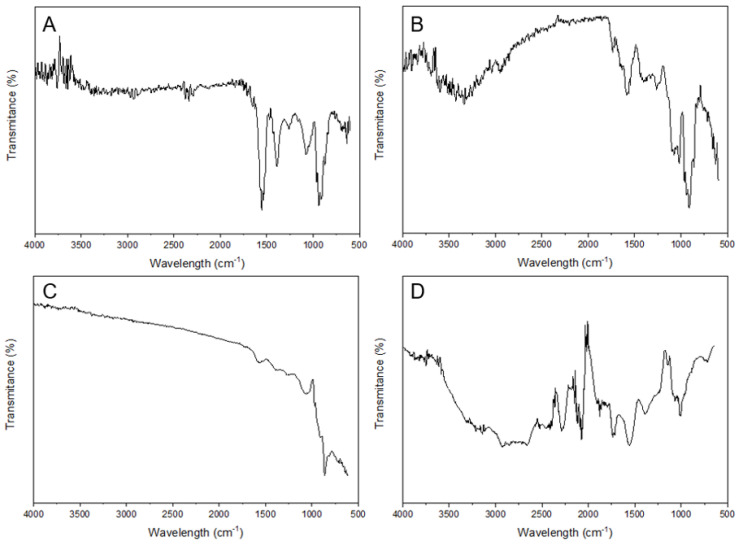
FT-IR spectra of the AgNPs synthesized using citrus peels extracts. (**A**) AgNPs synthesized with lemon extract. (**B**) AgNPs synthesized with orange extract. (**C**) AgNPs synthesized with lemon and grapefruit extract. (**D**) AgNPs synthesized with a mixture of lemon, orange, and grapefruit extracts.

The FTIR spectra of the silver nanoparticles synthesized with aqueous extracts of orange, lemon, grapefruit, and their mixture were analyzed to understand their chemical composition and functional groups [37]. In the lemon extract-synthesized silver nanoparticles (Figure 8A), the peaks observed at 639.28, 1168.6, and 1256.3 cm^−1^ are attributed to the stretching of C-C bonds, while the peak at 941.09 cm^−1^ corresponds to the stretching of C-H bonds. The peaks at 1555.3 and 1571.7 cm^−1^ are attributed to the stretching vibrations of C=O bonds, and the peak at 2299.6 cm^−1^ corresponds to the stretching of O-H bonds. In the case of orange extract-synthesized silver nanoparticles (Figure 8B), the peaks at 600.71 and 626.75 cm^−1^ correspond to the bending vibrations of C-H bonds, while the peak at 719.31 cm^−1^ is attributed to the stretching vibrations of C-H bonds. The peaks at 1024.0 and 1076.0 cm^−1^ are attributed to the stretching vibrations of C-O bonds, while the peak at 1406.8 cm^−1^ corresponds to the stretching vibrations of C-N bonds. In the grapefruit extract-synthesized silver nanoparticles (Figure 8C), the peaks observed at 614.21 and 1575.5 cm^−1^ correspond to the bending vibrations of C-H bonds, while the peak at 1394.2 cm^−1^ corresponds to the stretching vibrations of C-N bonds. In the case of the silver nanoparticles synthesized using a mixture of extracts (Figure 8D), the peaks observed at 749, 1068, and 1558 cm^−1^ correspond to the stretching vibrations of C-H bonds, while the peak at 1712 cm^−1^ corresponds to the stretching of C=O bonds. The peaks between 1840 and 2290 cm^−1^ are due to CO_2_ in the ambient environment [38], while the peaks between 2830 and 2923 cm^−1^ and 3100 to 3320 cm^−1^ correspond to the stretching vibrations of C-H and N-H bonds, respectively. The results show that each extract produces silver nanoparticles with unique functional groups and chemical compositions. These findings are useful in understanding the properties of silver nanoparticles synthesized using different natural extracts and their potential applications in various fields.

**Figure 9:**
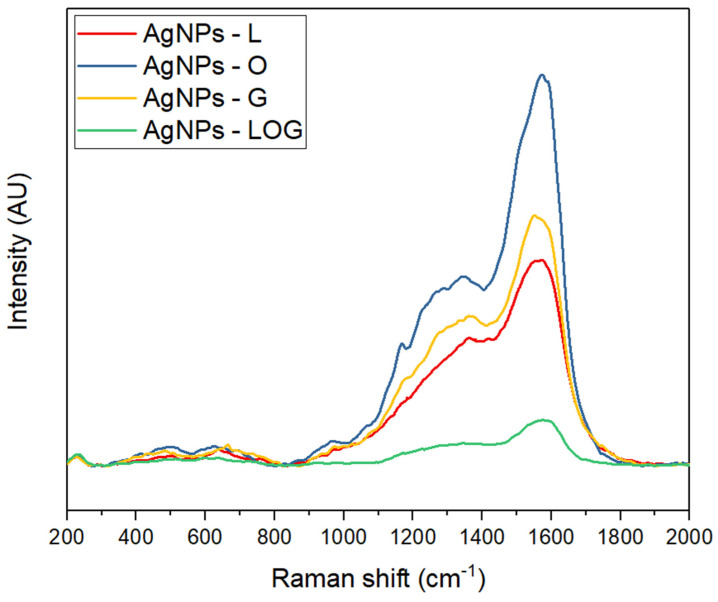
Raman spectra of the AgNPs synthesized using citrus peels extracts. (Red) AgNPs synthesized with lemon extract. (Blue) AgNPs synthesized with orange extract. (Yellow) AgNPs synthesized with lemon and grapefruit extract. (Green) AgNPs synthesized with a mixture of lemon, orange, and grapefruit extracts.

Raman spectroscopy was used to analyze the silver nanoparticles synthesized with aqueous extracts of orange, lemon, grapefruit, and their mixture (Figure 9). The spectra obtained showed distinctive peaks that correspond to the vibrational modes of the synthesized AgNPs. The peak at 226.44 cm^−1^ is attributed to Ag-S stretching, which confirms the presence of AgNPs. The peaks at 487.52, 662.52, and 961.62 cm^−1^ correspond to Ag-O stretching, which indicates the interaction of AgNPs with the functional groups present in the citrus extracts. The peak at 1165.41 cm^−1^ is associated with C-C stretching in polysaccharides, which confirms the presence of reducing agents in the extract. The peaks at 1280.98 and 1348.6 cm^−1^ correspond to C-H bending in aliphatic compounds and C-N stretching, respectively. The peak at 1568.66 cm^−1^ is attributed to N-H bending in amines, which indicates the presence of proteins or amino acids in the extract.

### 3.2. Antimicrobial Activity

**Figure 10:**
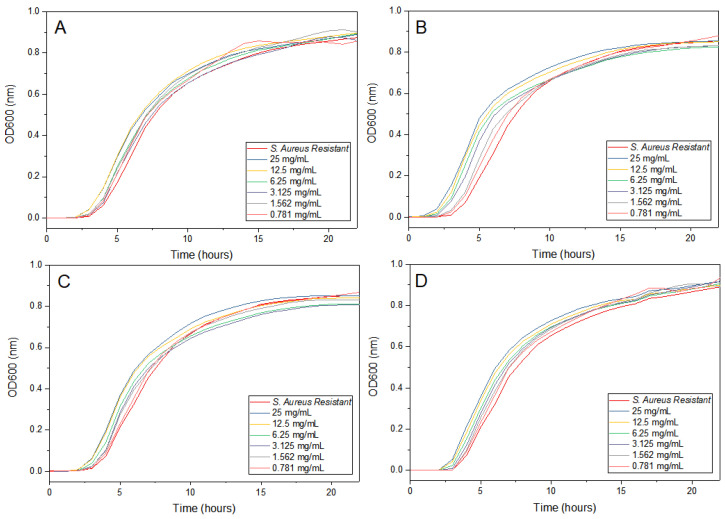
Antimicrobial activity of citrus peels extracts at different times. (**A**) Lemon peel extract. (**B**) Orange peel extract. (**C**) Grapefruit peel extract. (**D**) Mixture of lemon, orange, and grapefruit extracts.

**Figure 11:**
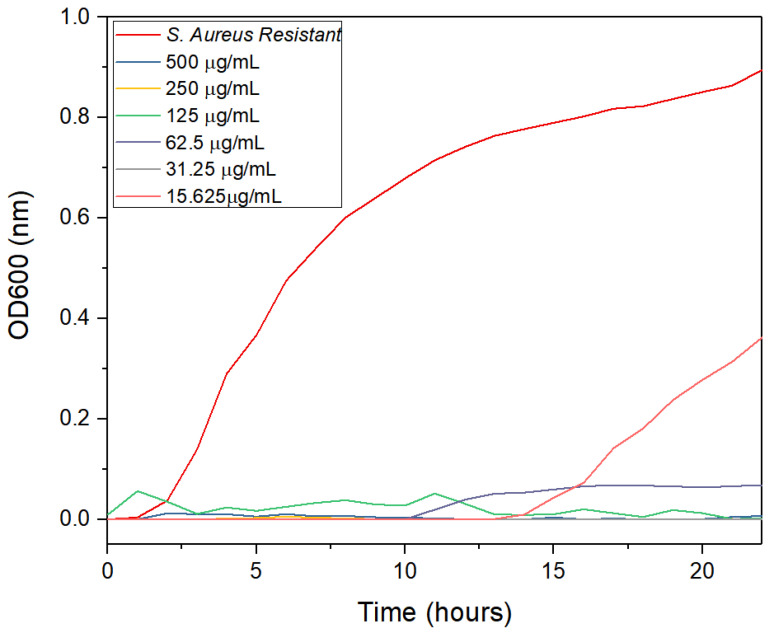
Antimicrobial activity of the AgNPs synthesized with lemon peel extract at different times.

**Figure 12:**
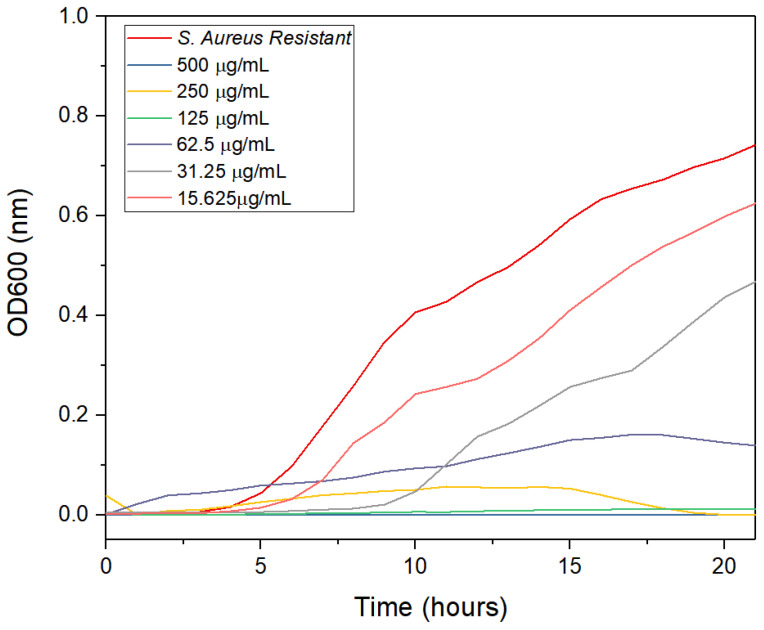
Antimicrobial activity of the AgNPs synthesized with orange peel extract at different times.

**Figure 13:**
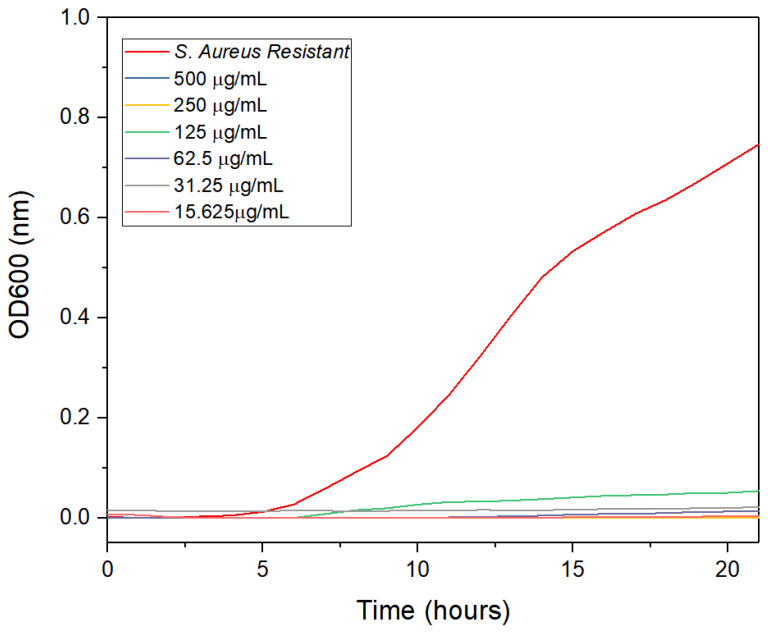
Antimicrobial activity of the AgNPs synthesized with grapefruit peel extract at different times.

**Figure 14:**
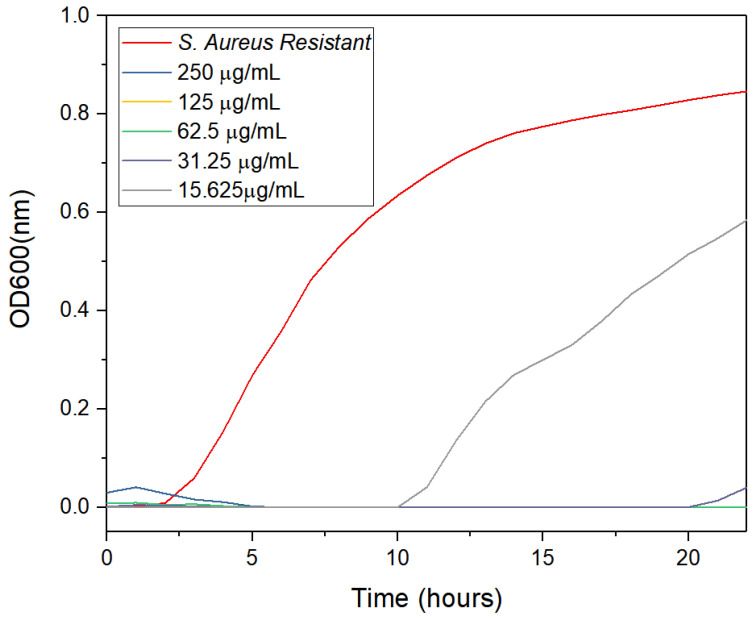
Antimicrobial activity of the AgNPs synthesized with lemon, orange, and grapefruit peel extract at different times.

The antibacterial activity of the synthesized AgNPs against clinical multidrug-resistant *Staphylococcus aureus* (SaR) was evaluated using the serial dilution method. As the concentration of AgNPs decreased, bacterial growth increased. However, the AgNPs synthesized with lemon peel extract and a combination of extracts exhibited the lowest concentration (15.625 μg/mL) at which the bacteria grew, while the AgNPs synthesized with orange peel extract showed inhibition at 62.5 μg/mL. Notably, the AgNPs synthesized with grapefruit peel extract exhibited no bacterial growth at all. The minimum inhibitory concentration (MIC) values of the citrus peel extracts and the AgNPs were determined over a 22-h interval, and the results are shown in Figure 10, Figure 11, Figure 12, Figure 13 and Figure 14. Overall, the results indicate that silver nanoparticles have a significant inhibitory effect on the growth of resistant *S. aureus*.

## 4. Discussion

### 4.1. Synthesis and Characterization of Ultra-Small AgNPs

It is known that the extracts of plants, fruits, and vegetables contain secondary metabolites such as flavonoids, phenolic acids, terpenoids, and alkaloids. These molecules contain hydroxyl groups which are used to carry out the redox reactions that are responsible for the synthesis of AgNPs since they are involved in the reduction of silver ions into metallic silver atoms which then agglomerate and grow to form nanostructures [39].

Much of the flavonoids in citrus are flavanones bound together as glycosides. In the orange and tangerine peel, hesperidin predominates; in grapefruit, naringin and narirutin stand out; finally, eriocitrin and hesperidin stand out in lemon and lime [40,41].

The synthesis of AgNPs using citrus peel extracts has been extensively studied due to their potential applications in various fields. In this study, the UV-Vis spectrum was used to confirm the presence of AgNPs in samples synthesized using an aqueous solution containing extracts of orange, lemon, grapefruit, or their mixture. Figure 1 shows the UV-Vis spectrum of the AgNPs synthesized in water, confirming that there was no reduction of the silver salt by itself. In contrast, the AgNPs synthesized with citrus peel extracts exhibit a plasmon resonance spectrum with an absorption band between 400 and 450 nm, indicating the presence of AgNPs. The UV-Vis spectrum of the extract of the mixture of the three citrus peels as of the ultra-small AgNPs shows no absorbance peak for the extract obtained. However, the ultra-small AgNPs show a maximum absorbance of 422 nm, reflecting the surface plasmon resonance of silver (Figure 5A). These results are consistent with previous studies that have reported similar maximum absorption peaks for AgNPs synthesized using grape extract or an aqueous extract of grape pomace [42,43]. The peak width of the AgNPs synthesized with grapefruit peel extract may be due to the variation in particle size. Similarly, the histograms in Figure 6C and Figure 7C show the particle size distribution of the AgNPs synthesized with citrus peel extracts, indicating that the size of the AgNPs varies with the type of extract used [44]. 

The TEM images presented in this study reveal that the synthesized AgNPs exhibit a spherical shape with a narrow size distribution. Interestingly, the orange extract-synthesized nanoparticles had an average size of 4.25 nm with a positively skewed histogram, indicating the presence of smaller nanoparticles, while the mixed extract-synthesized nanoparticles had an average size of 2.42 nm with a normal distribution histogram, indicating a relatively even distribution of sizes. These results are consistent with other reports in the literature that have utilized green synthesis methods to produce ultra-small AgNPs. For example, ultra-small AgNPs of sizes between 3 and 6 nm have been obtained with extracts of *Sida cordifolia* [34]; using *Mentha aquatica* leaf extract [36], sizes of 8 nm were obtained; and sizes of 0.5 to 1.4 nm were achieved using the extract of Rosa ‘*Andeli*’ [35].

The physical and chemical characterization of these ultra-small AgNPs gives a helpful guideline showing that aqueous extracts of lemon, orange, grapefruit, and a mixture of these citrus peels can carry out an adequate reduction of silver ions and, in addition, stabilize their surface [45].

The Scherrer equation and TEM imaging are both important tools for determining the size of nanoparticles. However, it is important to note that the Scherrer equation provides an estimate of the crystal size, while TEM imaging provides a more accurate measure of the particle size. This difference in measurement is due to the Scherrer equation providing an average size of the crystal, while TEM imaging measures the size of the individual particles.

In the SAED analysis, the clear and distinct circular patterns observed can be attributed to the (111), (200), (220), (311), (331), and (222) planes of the face-centered cubic (fcc) metallic silver, as confirmed by the Joint Committee on Powder Diffraction Standards file number 87-0720. At the same time, the EDS analysis confirms that the ultra-small AgNPs obtained are made up of metallic silver and evidences their crystalline nature (Figure 5D). Furthermore, similar results of EDS and SAED analyses of AgNPs have been reported when extracts of orange peel [46], lemon peel [47], and *Muntingia Calabura* [48] have been used.

The FTIR spectra of the silver nanoparticles synthesized using different citrus extracts showed unique functional groups and chemical compositions, which can be attributed to the presence of different organic compounds in each extract. In the case of silver nanoparticles synthesized using lemon extract, the observed peaks are attributed to the stretching of C-C and C-H bonds, while in the case of orange extract-synthesized silver nanoparticles, the peaks are attributed to the stretching vibrations of C-H, C-O, and C-N bonds. Similarly, grapefruit extract-synthesized silver nanoparticles showed peaks corresponding to the bending vibrations of C-H bonds and the stretching vibrations of C-N bonds. The results indicate that the choice of extract has a significant impact on the functional groups and chemical composition of silver nanoparticles.

Raman spectroscopy was also used to analyze the synthesized silver nanoparticles and the obtained spectra confirmed the presence of AgNPs. The peaks observed at 487.52, 662.52, and 961.62 cm^−1^ in the Raman spectra suggest the interaction of AgNPs with the functional groups present in the citrus extract. The peak at 1165.41 cm^−1^ confirms the presence of reducing agents in the extract, while the peaks at 1280.98 and 1348.6 cm^−1^ suggest the presence of aliphatic compounds and amines, respectively.

### 4.2. Antimicrobial Activity

The results presented in this study show that green-synthesized AgNPs have potential as effective antibacterial agents against multidrug-resistant *S. aureus*, whereas the extracts obtained alone do not have an antibacterial effect (Figure 10), confirmingthe antimicrobial effect of the the AgNPs obtained. The MIC values determined in this study (ranging from 15.625 µg/mL to 62.5 µg/mL) indicate that the nanoparticles have a bacteriostatic effect at lower concentrations and a bactericidal effect at higher concentrations [49]. This finding is consistent with previous studies in which AgNPs were found to inhibit bacterial growth by disrupting the cell membrane and producing reactive oxygen species. The unique characteristic of the ultra-small AgNPs used in this study is their size, which allows them to easily penetrate the cell wall of bacteria, resulting in a larger surface area and increased ROS production [50]. Bacterial growth can resume after AgNP treatment due to the presence of adaptations or mutations, or because the dormant bacteria may resume growth under favorable conditions.

Our study demonstrates that the MIC values of these ultra-small AgNPs are more efficient than those reported in previous studies (Figure 1), indicating that they have strong potential as antibacterial agents. Overall, our findings suggest that green-synthesized AgNPs, especially those with ultra-small particle size, could be used as an alternative strategy for treating multidrug-resistant bacterial infection. Further research is needed to explore the potential of these nanoparticles in clinical settings. In this regard, it is worth noting that the effective concentration 50 (EC_50_) of AgNPs in the literature is reported to be 5 μg/mL for a size of 4.7 nm and 2000 μg/mL for 42 nm [51].

As previously mentioned, the results obtained from the ultra-small AgNPs synthesized using the aqueous extracts of orange, lemon, grapefruit, and mixed peels showed superior antibacterial activity against resistant *S. aureus* strains compared to other investigations using AgNPs (Table 1). Additionally, the MICs of the extracts of lemon, orange, and grapefruit alone were included, which had much higher MIC values compared to the AgNPs. Moreover, in order to compare the MIC values of AgNPs with standard drugs, the data of the MICs of *S. aureus* strain used in this study for the antibiotics kanamycin and ampicillin, were also reported [6], showing a much greater antimicrobial activity by the AgNPs synthesized in our study, with respect to the drug kanamycin.

The ultra-small size of the AgNPs produced in our study resulted in a large surface area, to which their increased effectiveness against resistant *S. aureus* is attributed. Therefore, these nanoparticles could be used as an effective treatment for infections caused by resistant bacteria. Moreover, the synthesis method used in our study is environmentally sustainable as organic waste was used as a source of reducing and stabilizing extracts. In addition, no energy for extraction and synthesis was used, making it an even more sustainable process.

## 5. Conclusions

The utilization of citrus peel extracts in the green synthesis of ultra-small silver nanoparticles (AgNPs) has displayed remarkable potential as an effective antimicrobial agent against drug-resistant bacteria. The study findings suggest that AgNPs exhibit bacteriostatic effects at low concentrations and bactericidal effects at higher concentrations, positioning them as an attractive alternative to conventional antibiotics. The ultra-small size of AgNPs, their strong penetration properties, and their larger surface area provide them with an advantage in crossing the bacterial cell wall and generating reactive oxygen species that affect cellular function, thus augmenting their antimicrobial activity.

Furthermore, the use of citrus peels as a source of reducing and stabilizing agents in the synthesis of AgNPs represents an eco-friendly and sustainable approach. It offers numerous benefits, such as the valorization of citrus waste, the use of less toxic solvents and reagents, a reduction in energy consumption and process steps, and a substantial contribution to environmental preservation.

The green synthesis of AgNPs using citrus peels provides a sustainable solution for the citrus industry, as it employs the abundant waste produced by these fruits. This study demonstrates more efficient minimum inhibitory concentration (MIC) than existing antimicrobial agents, rendering it a valuable strategy to combat drug-resistant bacterial infections while also contributing to environmental protection.

## Figures and Tables

**Table 1:** *S. aureus* MICs from different sources.

Reference	Strain	Treatment	MIC (ppm)	Extract
[7]	*S. aureus* ATCC 6538	AgNPs	125	*Gardenia jasminoides* leaves
Padilla-Cruz, A. L. et al., 2021 [7]	*S. aureus* ATCC 29,213	AgNPs	125	*Gardenia jasminoides* leaves
Padilla-Cruz, A. L. et al., 2021 [7]	Multidrug-resistant *S. aureus*	AgNPs	125	*Gardenia jasminoides* leaves
[39]	*S. aureus*	AgNPs	50	*Galega officinalis*
Garza-Cervantes, J. A. et al., 2020 [6]	*S. aureus*-Kan	Kanamycin	256	NA
Garza-Cervantes, J. A. et al., 2020 [6]	*S. aureus*-Amp	Ampicillin	1	NA
[52]	*S. aureus*	Extract	14,000	Lemon peel
Razmjoo M. et al., 2016 [53]	*S. aureus*	Extract	15,000	Orange peel
Arsène, M et al., 2021 [54]	*S. aureus* ATCC 6538	Extract	6250	Grapefruit peel
This work	Multidrug-resistant *S. aureus*	AgNPs	31.25	Lemon peel
This work	Multidrug-resistant *S. aureus*	AgNPs	62.50	Orange peel
This work	Multidrug-resistant *S. aureus*	AgNPs	15.625	Grapefruit peel
This work	Multidrug-resistant *S. aureus*	AgNPs	15.625	Orange lemon and grapefruit peels

## Data Availability

All data generated or analyzed during this study are included in this published article.
